# Supplementary figures and images for: The effects of plantarflexor weakness and reduced tendon stiffness with aging on gait stability
Source: PLoS One. 2024 Apr 16;19(4):e0302021. doi: 10.1371/journal.pone.0302021 (PMC11020829; doi:10.1371/journal.pone.0302021)

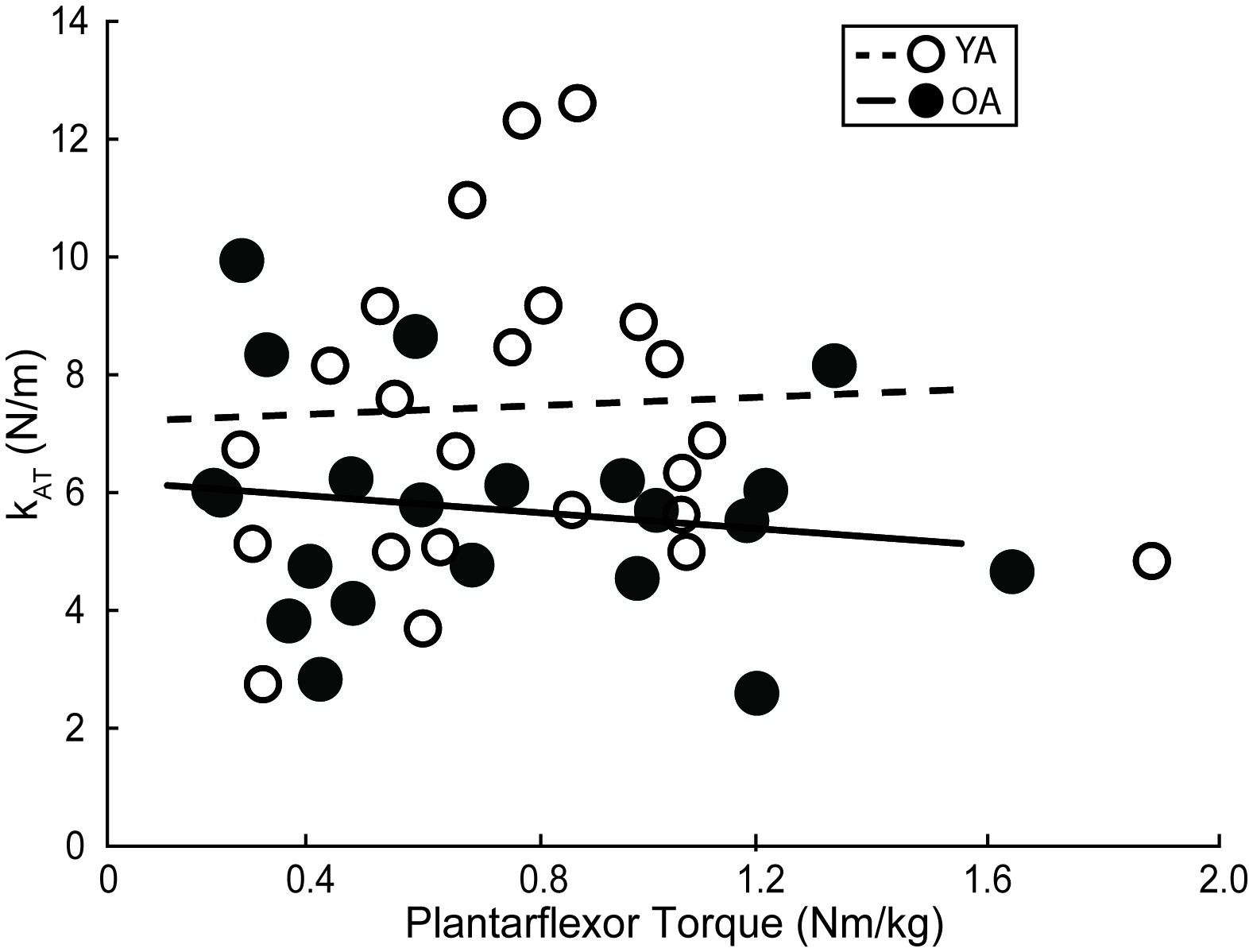

Supplement: S1 Appendix — Individual average peak plantarflexor torque and Achilles tendon stiffness (kAT) for older (solid line, n = 21) and younger (dashed line, n = 22) adults. Lines of best fit for both groups are shown and significant correlations are depicted per group by their corresponding r-value accompanied by an asterisk. (p = 0.05). (TIF) [file pone.0302021.s001.tif]
